# Draft genome sequence of *Dietzia* sp. strain CH92 isolated from oil reservoir

**DOI:** 10.1128/mra.01065-23

**Published:** 2024-02-01

**Authors:** Wei Xiang, Quan Zhang, Jianxin Wang, Yanfen Xue, Bo Yu

**Affiliations:** 1 SINOPEC (Dalian) Research Institute of Petroleum and Petro-Chemicals Co., Ltd., Dalian, China; 2 Institute of Microbiology, Chinese Academy of Sciences, Beijing, China; 3 University of Chinese Academy of Sciences, Beijing, China; University of Southern California, USA

**Keywords:** *Dietzia* sp., hydrocarbons, degradation

## Abstract

We report the draft genome sequence of *Dietzia* sp. strain CH92, isolated from a high temperature oil well in Baolige oilfield, China. The estimated genome is 3.73 Mb, with 3,479 protein-coding sequences.

## ANNOUNCEMENT

*Dietzia* species are aerobic, Gram-positive and distributed in various environments including crude oil reservoirs (1, 2). The organisms can use carbohydrates, organic acids, and hydrocarbons as carbon and energy sources (2). *Dietzia* sp. strain CH92 was isolated from oil-well production liquid of Baolige oilfield (44°86′N, 115°81′E) in China. The whole-genome sequence will help us understand its function and application potential.

Strain CH92 was isolated by dilution plating method. The oil-well production liquid sample collected from a sampling valve at the pipeline of the well head by fully filling 25 L plastic sampling bottles. The samples were transported to the laboratory and stored overnight at ambient temperature (about 15–20°C). Since the cell biomass is rather low in this water sample, we first concentrated it by using 0.22 µm hollow-fiber filter (MOF-1d, purchased from Motech Co. Ltd., Tianjin, China), and then, the filtrate was used for dilution and plating on the agar medium. The concentrated liquid was diluted with sterilized enrichment medium (ENM), plated on ENM agar, and incubated at 45°C (3). The pure strain was obtained by repeated streaking on the same medium agar plates. Strain CH92 was routinely cultured at 37°C in modified DSMZ 878 medium (4) supplemented with 10 g L^−1^ succinate or 1% (wt/vol) *n*-hexadecane as carbon sources. Genomic DNA was prepared from an overnight culture in the modified DSMZ 878 medium with the addition of g L^−1^ succinate by using the TIANamp Bacteria DNA kit (TIANGEN, China). The quality and concentration of DNA were determined using a Quantus Fluorometer with the Quant-iT PicoGreen dsDNA Assay Kit (Thermo Fisher Scientific, USA).

DNA samples were sheared into 400–500 bp fragments using a Covaris M220 Focused Acoustic Shearer following manufacture’s protocol. The Illumina PE libraries were prepared from the sheared fragments using the NEXTflex Rapid DNA-Seq Kit (Bioo Scientific, USA) and sequenced in the 150 bp pair-end mode using the Illumina Hiseq × 10 platform at Majorbio Bio-Pharm Technology Inc. (Shanghai, China). The sequencing generated 5,876,037 pairs of raw reads totaling 1,774,563,174 bp, giving approximately 473 × coverage. The reads were quality-trimmed with Trimmomatic v.0.36 (5) and assembled using SOAPdenovo v2 (6). The resultant assembly totaled 3,729,715 bp with 32 contigs, an N50 value of 636,736 bp, and a GC content of 71.40%.

The genomic contigs were analyzed using I-Sanger Cloud Platform from Shanghai Majorbio in March, 2021. Glimmer v3.02 (http://ccb.jhu.edu/software/glimmer/index.shtml) (7) was used for coding DNA sequence (CDS) prediction, tRNA-scan-SE v2.0 (http://trna.ucsc.edu/software/) (8) was used for tRNA prediction, and Barrnap v0.8 (https://github.com/tseemann/barrnap) was used for rRNA prediction. The predicted CDSs were annotated from NR, Swiss-Prot, Pfam, GO, COG, and KEGG database using sequence alignment tools: BLAST + v2.3.0 (9), Diamond v0.8.35 (10), and HMMER v3.1b2 (11). A total of 3,479 CDS genes in addition to 50 tRNAs and 5 rRNAs were annotated for the draft genome sequence. Default parameters were used for all software. The annotation was also uploaded in Fig Share with the link of https://figshare.com/articles/online_resource/The_genome_annotation_with_predicted_functions_for_each_and_every_gene_of_i_Dietzia_i_sp_strain_CH92/24967986.

## Data Availability

This whole-genome shotgun sequencing project has been deposited in DDBJ/ENA/GenBank under the accession number JAVHXC000000000; the raw reads are available under SRA accession number SRR24848572. This announcement represents the first version of the genome.
